# Long noncoding RNA HOTAIR regulates the invasion and metastasis of prostate cancer by targeting hepaCAM

**DOI:** 10.1038/s41416-020-01091-1

**Published:** 2020-10-07

**Authors:** Ting Li, Nanjing Liu, Yingying Gao, Zhen Quan, Yanni Hao, Chaowen Yu, Luo Li, Mengjuan Yuan, Lingfang Niu, Chunli Luo, Xiaohou Wu

**Affiliations:** 1 Key Laboratory of Laboratory Medical Diagnostics, Ministry of Education, Chongqing Medical University, 400016 Chongqing, China; 2 Center for Clinical Molecular Medicine; Chongqing Key Laboratory of Pediatrics; Ministry of Education Key Laboratory of Child Development and Disorders; National Clinical Research Center for Child Health and Disorders; China International Science and Technology Cooperation Base of Child Development and Critical Disorders, Children’s Hospital of Chongqing Medical University, 400016 Chongqing, China; 3 Department of Urology, The First Affiliated Hospital of Chongqing Medical University, 400016 Chongqing, China

**Keywords:** Long non-coding RNAs, Prostate cancer, Gene silencing

## Abstract

**Background:**

The role of HOX transcript antisense RNA (HOTAIR) has been proven to be important in tumorigenesis. However, how this molecule promotes metastasis and invasion in PCa is still unclear.

**Methods:**

The relationship between HOTAIR and hepatocellular adhesion molecule (hepaCAM) in PCa was identified by immunohistochemistry, immunofluorescence, plasmid transfection, quantitative real-time PCR and immunoblotting. The regulatory effects of HOTAIR on hepaCAM and MAPK signalling and their key roles in PCa metastasis were investigated in vitro.

**Results:**

The expression of HOTAIR was inversely correlated with hepaCAM in the blood and tissue of PCa patients. Here, hepaCAM was identified as a novel target gene of HOTAIR and was critical for the invasiveness of PCa. HOTAIR recruited PRC2 to the hepaCAM promoter, resulting in high levels of H3K27me3 and the absence of hepaCAM with an abnormally activated MAPK pathway. Both HOTAIR depletion and EZH2 inhibition could induce hepaCAM re-expression with inhibitory MAPK signalling and decrease the invasive and metastatic capabilities of PCa cells.

**Conclusions:**

This study demonstrates that HOTAIR promotes invasion and metastasis of PCa by decreasing the inhibitory effect of hepaCAM on MAPK signalling. Therefore, the HOTAIR/hepaCAM/MAPK axis may provide a new avenue towards therapeutic strategies and prognostic indicators for advanced prostate cancer.

## Background

Prostate cancer (PCa) remains one of the most common malignant tumours in men. Due to the early mild symptoms and occult incidence, most cases are diagnosed in the advanced stages with metastasis.^1^ Despite numerous advances in PCa treatment, docetaxel remains ineffective in improving the prognosis of patients with metastasis. To improve outcomes, numerous studies have evaluated docetaxel in combination with other agents.^2^ Exploring the potential mechanism that promotes PCa metastasis will help identify novel prognostic markers or therapeutic targets. With the deepening of tumour transcriptomics, long noncoding RNAs (lncRNAs) have been confirmed to be associated with the occurrence and metastasis of tumours.^3^ Some aberrantly expressed lncRNAs have been identified in PCa cell lines and clinical samples and may play critical roles in the pathogenesis of PCa.

HOX transcript antisense RNA (HOTAIR) is a long intergenic noncoding RNA that is closely related to tumour metastasis and poor recurrence-free survival.^4,5^ Moreover, studies have identified HOTAIR as a novel diagnostic and prognostic biomarker.^6,7^ HOTAIR can recruit lysine-specific demethylase 1 (LSD1) and polycomb repressive complex 2 (PRC2). PRC2 is a histone methyltransferase complex composed of EZH2, SUZ12 and EED.^8,9^ Moreover, 3-deazaneplanocin A (DZNep), an inhibitor targeting PRC2, could inhibit the activity of S-adenosyl-L-homocysteine hydrolase.^10^ DZNep inhibits tumour characteristics,^11,12^ and its synergistic interaction with gemcitabine enhances tumour regulatory effects.^13^ However, the specific mechanisms underlying the metastatic regulation of HOTAIR in PCa are largely unclear. Therefore, the purpose of this study was to elucidate the role of HOTAIR in PCa and to provide alternative strategies for the development of targeted treatment and individualised treatment.

In this study, we demonstrated that elevated HOTAIR promotes PCa metastasis by targeting an immunoglobulin-like cell adhesion molecule: hepatocellular adhesion molecule (hepaCAM). HepaCAM is a transmembrane glycoprotein, and the extracellular region plays an important role in regulating cell adhesion and movement.^14,15^ Previous works have shown that hepaCAM is expressed in normal tissues but undetectable in cancer tissues. When wild-type hepaCAM was re-expressed, tumour characteristics were notably weakened.^16–18^ However, the mechanism of hepaCAM silencing and how it affects PCa metastasis remain poorly understood.

Accumulating evidence shows that the abnormal activation of some signalling pathways gives rise to tumour spread via disordered gene expression.^19^ The MAPK pathway is verified to mediate signal transduction and regulate cellular activities.^20,21^ Here, we showed that HOTAIR could inhibit hepaCAM transcription, causing uncontrolled metastasis via the MAPK pathway. These discoveries indicate that HOTAIR could serve as an attractive target for the treatment of docetaxel-insensitive prostate cancer. We envision that an epigenetic strategy combined with established chemotherapy is highly likely to improve treatment outcomes for these common male cancers.

## Methods

### Patients and specimens

Seventy PCa surgical specimens, 25 samples of benign prostatic hyperplasia (BPH) and the corresponding anti-coagulant blood samples (3–4 ml) were collected from patients in the Department of Urology at the First Affiliated Hospital of Chongqing Medical University (China) between 2016 and 2018. Written consent for the biological studies was obtained from the patients or their guardians. According to the WHO standard, each specimen was histologically examined and graded according to the UICC guidelines. Specimens were stored in liquid nitrogen before experiments.

### Immunohistochemistry

The sections were subjected to antigen retrieval, goat serum block, primary antibody incubation (hepaCAM: 1:300, Proteintech, Wuhan, China; H3K27me3: 1:500, Cell Signaling Technology (CST), MA, USA) overnight at 4 °C and secondary antibody incubation for 1 h at 37 °C. Visualisation was carried out by diaminobenzidine, and counterstaining was performed by haematoxylin. ImageJ software was used to analyse the staining intensity and positive rate score. Tumour and BPH tissues were categorised as positive and negative according to whether stained cells were ≥5%.

### Blood sampling and PBMC extraction

PBMCs in blood were isolated within 2 h by Ficoll (TBD Science, Tianjin, China) gradient centrifugation and were transferred into 1 ml of TRIzol Reagent (Invitrogen, Thermo Fisher Scientific, MA, USA) in 1.5 ml centrifuge tubes and stored at −80 °C until RNA extraction.

### Cell culture, treatment and transfection

RWPE-1, PC3 and DU145 cells were purchased from the American Type Culture Collection (ATCC). All experiments were performed with mycoplasma-free cells. All cells were cultured in RPMI-1640 (Gibco, Thermo Fisher Scientific, MA, USA) supplemented with 10% FBS (Gibco, Thermo Fisher Scientific) and 100 U/mL penicillin/streptomycin (Beyotime Institute of Biotechnology, Shanghai, China). Cell lines were cultured at 37 °C in a 5% CO_2_ humidified atmosphere. Adenoviruses containing green fluorescent protein (Ad-GFP) or hepaCAM (Ad-hepaCAM) were constructed by our group as previously described.^18^ The sgRNAs targeting HOTAIR were designed using the online tool available at www.crispr.mit.edu. The sequences of the sgRNAs were as follows: sh223-sg-HOTAIR#1 sense, 5′-ACCGGGGCCGCCCTCCTAGTGGTT-3′ and antisense, 5′-AAACAACCACTAGGAGGGCGGCCC-3′; sh223-sg-HOTAIR#2 sense, 5′-ACCGGAGGGGACGCACGTGTACC-3′ and antisense, 5′-AAACGGTACACGTGCGTCCCCTC-3′; sh223-sg-HOTAIR#3 sense, 5′-ACCGGCGGCTCTCGCCTGAGAAC-3′ and antisense, 5′-AAACGTTCTCAGGCGAGAGCCGC-3′; CAS9-Ctrl sense, 5′-ACCGGTTCTCCGAACGTGTCACGT-3′ and antisense, 5′-AAACACGTGACACGTTCGGAGAAC-3′. The CRISPR/Cas9 plasmids were constructed by Sangon Biotech (Shanghai, China). HepaCAM-targeting siRNA was purchased from GenePharma (Shanghai, China) and transfected into cells using Lipofectamine 2000 according to the manufacturer’s instructions (Invitrogen, Thermo Fisher Scientific). Other involved reagents were as follows: dimethyl sulfoxide (Sigma-Aldrich, CA, USA), 3-deazaneplanocin A (DZNeP, 1 µM, Selleck, USA), CPI-169 racemate (70 nM, Medchem Express, NJ, USA), D-erythro sphingosine-1-phosphate (S1P, 0.3 µM, Abcam, UK) and docetaxel (5 nM, Selleck).

### Reverse transcription and quantitative real-time PCR

Total RNA of tissue specimens, blood samples and cell lines was extracted using TRIzol (TaKaRa, Tokyo, Japan), and reverse transcription was performed by the Prime Script RT reagent kit (TaKaRa) according to the manufacturer’s protocols. Real-time PCR was performed with the SYBR Premix Ex Taq™ II kit (TaKaRa). The expression of genes was determined by the comparative 2 −▵▵CT method. Real-time PCR was performed in triplicate, and the results were normalised against β-actin. The sequences of the primers were as follows: hepaCAM sense, 5**′**-TGTACAGCTGCATGGTGGAGA-3**′** and antisense, 5**′**-TCTGGTTTCAGGCGGTCATCA-3**′**; HOTAIR sense, 5**′**-GGTAGAAAAAGCAACCACGAAGC-3**′** and antisense, 5**′**-ACATAAACCTCTGTCTGTGAGTGCC-3**′**; MMP-2 sense, 5**′**-TGGCAAGGAGTACAACAGC-3**′** and antisense, 5**′**-TGGAAGCGGAATGGAAAC-3**′**; MMP-9 sense, 5**′**-TCCCTGGAGACCTGAGAACC-3**′** and antisense, 5**′**-CGGCAAGTCTTCCGAGTAGTTT-3**′**; fibronectin sense, 5**′**-CTTACCCGAAGAGGAC-3**′** and antisense, 5**′**-AAGTTTGTTGGTGGAGA-3**′**; VEGF sense, 5**′**-GCTGTTCTCGCTTCGG-3**′** and antisense, 5**′**-TGATGATTCTGCCCTCCT-3**′**; and β-actin sense, 5**′**-TGACGTGGACATCCGCAAAG-3**′** and antisense, 5**′**-CTGGAAGGTGGACAGCGAGG-3**′**.

### Western blotting assay

Total protein of surgical specimens and cells was extracted using RIPA buffer containing protease inhibitor and phosphatase inhibitors, and the protein concentration was detected by a BCA Protein Assay kit (Beyotime Institute of Biotechnology). Protein samples separated by SDS-PAGE were transferred to PVDF membranes (Millipore, MA, USA). After the membranes were blocked, they were incubated with primary antibodies overnight and horseradish peroxidase-conjugated secondary antibodies for 2 h at room temperature. The intensity level of the protein bands was evaluated using Image‑Pro Plus 6.0 with an enhanced chemiluminescent kit (Millipore).

### Transwell and wound healing assays

For transwell invasion assays, 3 × 10^4^ cells were implanted in the upper chamber with a mixed Matrigel-coated membrane and incubated with FBS-free medium. Medium supplemented with FBS was added to the lower chamber. After 36 h of incubation, the invasive cells were stained with crystal violet, and five visual areas were randomly counted at 100-fold magnification under a microscope (Nikon, Tokyo, Japan). For the wound healing assay, 5 × 10^4^ cells per well were seeded into a 6-well plate. After 70% confluence was reached, the cells were wounded with a 200 µL pipette tip and cultured for another 24 h. Wound healing was observed under a microscope at the indicated time points.

### Colony formation assay

Four hundred treated cells were seeded in the wells of six-well plates. After 14 days of culture, the cells were washed with PBS and fixed with 4% paraformaldehyde for 10 min. The colonies were stained with crystal violet solution for 10 min. The number of colonies was counted using a light microscope. The colony formation assay was performed in triplicate.

### Immunofluorescence

Differently treated cells were reseeded on polylysine-coated coverslips and cultured for 18 h. The cells were washed with cold PBS, fixed in 4% paraformaldehyde, permeabilised with 0.1% Triton X-100 and blocked with 5% BSA containing 1% Tween-20. Immunostaining was performed with primary antibodies and corresponding secondary antibodies diluted in blocking buffer. Immunofluorescence images were captured by microscopy at ×400 magnification (Nikon).

### Chromatin immunoprecipitation (ChIP)

PC3 cells were crosslinked with 1% formaldehyde and then DE crosslinked with glycine. Chromatin digestion to a mean length of 500 bp and centrifugation were performed. The chromatin fragments were mixed with SUZ12, EZH2, H3K27me3 and control antibodies and then incubated overnight at 4 °C with rotation. After DNA purification and elution, RT-PCR was used to analyse the DNA enrichment.

### Coimmunoprecipitation assay

Co-IP was performed by a Thermo Scientific Pierce Co-IP kit (Thermo Scientific, MA, USA) following the manufacturer’s instructions. Briefly, 10 μg anti-H3K27me3 antibody (CST) and matched IgG were immobilised for 2 h by AminoLink Plus coupling resin. Then, the resin was washed with wash solution and incubated with 250 μg PC3 cell lysate at 4 °C overnight. After incubation, the resin was washed again, and the protein was eluted using elution buffer.

### Flow cytometry assay

PC3 cells were subjected to docetaxel (4 nM, Selleck) until 60% confluence and incubated for 48 h. For the apoptosis assay, cells were collected and subjected to flow cytometry. For the cell cycle assay, cells were collected and fixed with 75% ethanol, and then the cell cycle distribution was analysed by flow cytometry.

### Dataset analysis and code availability

Two gene datasets were applied: The UCSC Genome Browser (https://genome.ucsc.edu/) and the Ualcan (http://ualcan.path.uab.edu). All available gene expression data were downloaded. For identification of a population of PCa samples, all prostate cancer cases in TCGA datasets were grouped into populations. The positive population included samples with high levels of gene expression.

### Statistical analysis

Statistical analyses in this study were conducted using SPSS software, version 18.0 (IL, USA). All experiments were conducted at least three times, and the data are represented as the mean ± SD. Student’s *t*-test, Chi-square test, one-way ANOVA, two-way ANOVA and Spearman analysis were used to evaluate the significant associations among categorical variables. *P*-values  < 0.05 were considered statistically significant.

## Results

### HOTAIR is highly expressed in the tissue and blood of prostate cancer patients and is correlated with the levels of H3K27me3 and hepaCAM

**Fig. 1:**
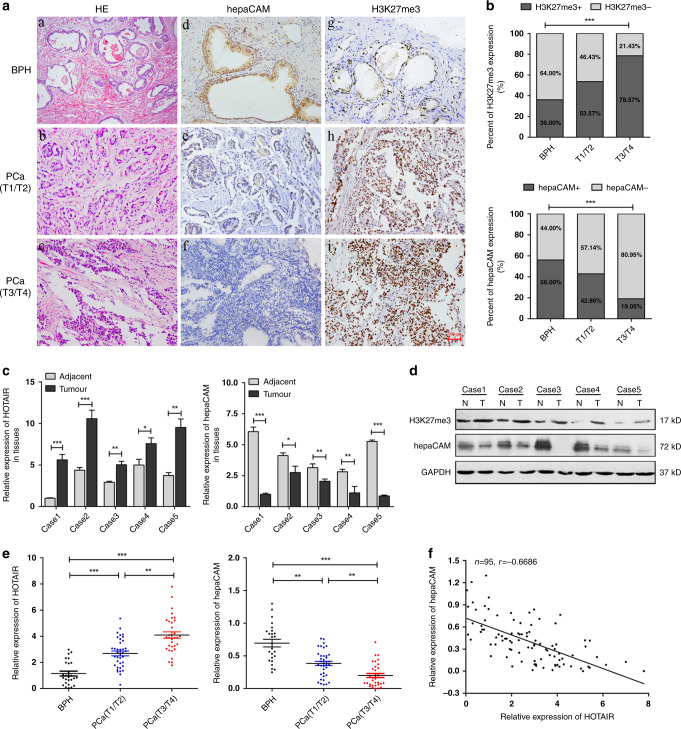
HOTAIR is highly expressed in the tissue and blood of PCa patients and is correlated with the levels of H3K27me3 and hepaCAM. **a** Representative IHC images. **a**(a–c) Identification of PCa and BPH tissues by HE staining. **a**(d–f) HepaCAM staining intensity in BPH and PCa tissues. **a**(g–i) H3K27me3 staining in BPH and PCa tissues. **b** Higher percentage of H3K27me3 staining and lower percentage of hepaCAM staining in PCa tissues; ***P < 0.001. **c**, **d** The molecular expression level in fresh prostate tumour specimens (T) and paired adjacent normal tissues (N); *P < 0.05, **P < 0.01, ***P < 0.001. **e** The altered expression of HOTAIR and hepaCAM in the blood of patients with PCa at different T stages; **P < 0.01, ***P < 0.001. **f** Negative correlation between HOTAIR and hepaCAM in PCa patient blood; *r* = −0.6686.

The collected tissue specimens were confirmed by H&E staining (Fig. 1a(a–c)). The expression of hepaCAM and H3K27me3 in 70 PCa specimens and 25 benign prostatic hyperplasia (BPH) samples was examined by immunohistochemistry (IHC). HepaCAM staining was high in the cytomembrane and cytoplasm in the BPH cells (Fig. 1a(d)) but was low or absent in the PCa cells (Fig. 1a(e, f)). As shown in Fig. 1a(g–i), H3K27me3 staining was rarely observed in the BPH tissues but was mainly concentrated in the nucleus of PCa cells. The IHC staining intensity detected by ImageJ revealed that H3K27me3 in the tumour tissues was significantly higher than that in the BPH tissues, and the intensity was increased with carcinoma progression, while hepaCAM showed the opposite pattern (Fig. 1b).

In the PCa group, 15 (53.57%) stage T1/T2 cases and 33 (78.57%) stage T3/T4 cases were positive for H3K27me3 (H3K27me3+), while 13 (46.43%) stage T1/T2 cases and 9 (21.43%) stage T3/T4 cases were negative for H3K27me3 (H3K27me3-). HepaCAM was positively expressed (hepaCAM+) in 12 (42.86%) stage T1/T2 cases and 8 (19.05%) stage T3/T4 cases but negatively expressed (hepaCAM−) in 16 (57.14%) stage T1/T2 cases and 34 (80.95%) stage T3/T4 cases. In the BPH group, 16 (64.00%) cases were H3K27me3-, but only 9 (36.00%) cases were H3K27me3+, and 56% were hepaCAM+, while 44% were hepaCAM− (Fig. 1b).

The relationship between the expression of H3K27me3 and hepaCAM in tissues and clinicopathological parameters was then analysed. These data showed that H3K27me3 expression was positively correlated with T stage (*p* = 0.0273, Table 1) and bone metastasis (*p* = 0.0280, Table 1), while hepaCAM was negatively correlated with tumour T stage (*p* = 0.0308, Table 1) and bone metastasis (*p* = 0.0435, Table 1). Thus, higher H3K27me3 and lower hepaCAM were expressed in advanced PCa patients, indicating that there may be a potential relationship between them.

**Table 1:** The correlation between H3K27me3/hepaCAM expression and clinical pathologic parameters of PCa.

Clinicopathological parameter	Total no.	H3K27me3		hepaCAM		*p* values	
		Negative (%)	Positive (%)	Negative (%)	Positive (%)	H3K27me3	hepaCAM
Age (years)						0.3333	0.2482
<60	21	8 (38)	13 (62)	17 (81)	4 (19)		
≥60	49	13 (27)	36 (73)	33 (67)	16 (33)		
Histological stage						**0.0273**	**0.0308**
T1/T2	28	13 (46)	15 (54)	16 (57)	12 (43)		
T3/T4	42	9 (21)	33 (79)	34 (81)	8 (19)		
Lymph node metastasis						0.1734	0.5439
Absent	38	14 (37)	24 (63)	26 (68)	12 (32)		
Present	32	7 (22)	25 (78)	24 (75)	8 (25)		
Bone metastasis						**0.0280**	**0.0435**
Absent	43	17 (40)	26 (60)	27 (63)	16 (37)		
Present	27	4 (15)	23 (85)	23 (85)	4 (15)		
Histological grade						0.8076	0.8032
Low	29	10 (34)	19 (66)	18 (62)	11 (38)		
High	41	13 (32)	28 (68)	27 (66)	14 (34)		

Numbers in bold font indicate statistical significance.

In five fresh PCa surgical specimens and the corresponding adjacent normal tissues, the HOTAIR mRNA and H3K27me3 protein levels were significantly higher in the tumour tissues (T) than in the adjacent normal tissues (N), while the mRNA and protein levels of hepaCAM showed the opposite pattern (Fig. 1c, d).

To further evaluate the role of HOTAIR in PCa, we measured the relative expression of HOTAIR and hepaCAM in blood samples from 70 patients with PCa and 25 patients with BPH. Compared to the patients with BPH, the PCa patients exhibited increased expression of HOTAIR and decreased hepaCAM (Fig. 1e). The following data analysis showed a significantly negative correlation between HOTAIR and hepaCAM expression in the blood of the PCa patients (*R* = −0.6686, Fig. 1f). These results indicated that HOTAIR and hepaCAM were involved in PCa progression and might act as prognostic biomarkers.

### Silencing HOTAIR in PCa cells significantly upregulates hepaCAM levels

**Fig. 2:**
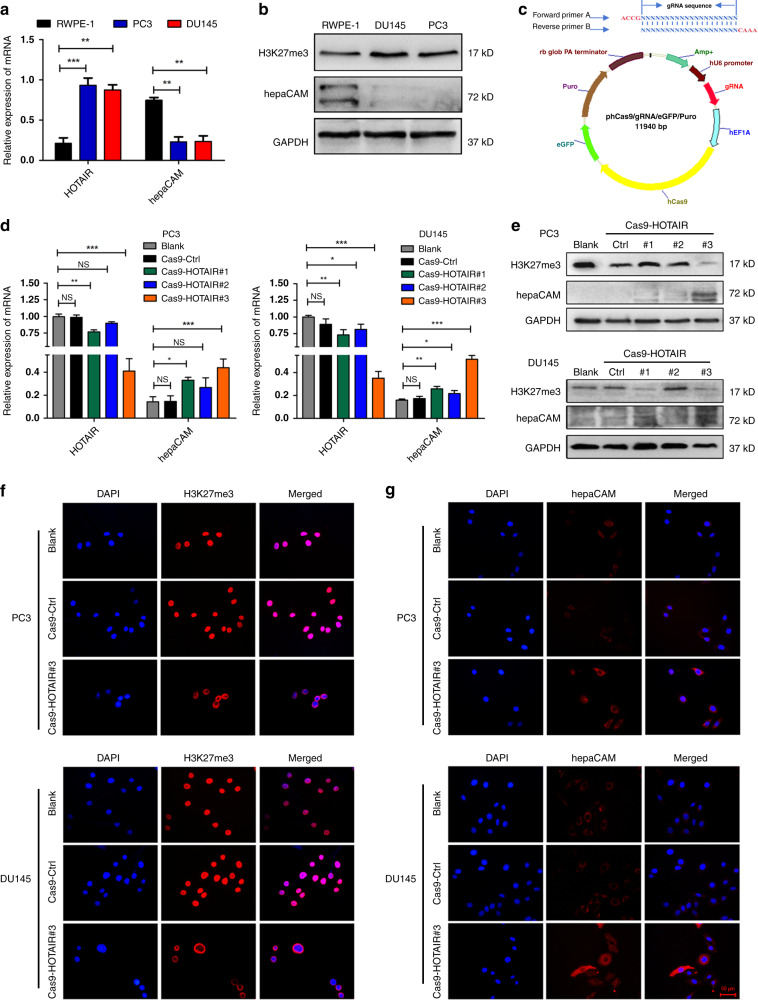
Silencing HOTAIR in PCa cells significantly upregulates hepaCAM levels. **a**, **b** RT-PCR and western blotting demonstrating higher HOTAIR and H3K27me3 and lower hepaCAM levels in PCa cells compared to PWPE-1 cells; **P < 0.01, ***P < 0.001. **c** Construction of the CRISPR/Cas9 plasmid. **d**, **e** Verification of HOTAIR knockout and hepaCAM re-expression effects of the Cas9-plasmid by RT-PCR. Data are shown as the mean ± SD (*n* = 3), NS, not significant, *P < 0.05, ***P < 0.001. **f**, **g** Knockout of HOTAIR can change the nuclear localisation of H3K27me3, thereby losing the inhibitory effect on hepaCAM.

Consistent with the tissue expression, HOTAIR and H3K27me3 were highly expressed in PCa cells compared with RWPE-1 human prostate epithelial cells. However, hepaCAM expression at the mRNA and protein levels in the PCa cell lines was lower than that in the RWPE-1 cells (Fig. 2a, b). We silenced HOTAIR with the CRISPR/Cas9 plasmid in PC3 and DU145 cells (Fig. 2c). Compared with Cas9-Ctrl, Cas9-HOTAIR#1 and #2, Cas9-HOTAIR#3 significantly downregulated the expression of HOTAIR; hence, Cas9-HOTAIR#3 was used for further experiments (Fig. 2d). Loss of HOTAIR significantly reduced the expression of H3K27me3 and upregulated the expression of hepaCAM (Fig. 2d-g) in PCa cells. In addition, H3K27me3 was transferred from the nucleus to the nuclear membrane, losing its regulatory function at the chromatin level, and the suppressor gene hepaCAM was re-expressed (Fig. 2f, g).

### HOTAIR deficiency strongly reduces the invasiveness and metastasis of PCa cells

**Fig. 3:**
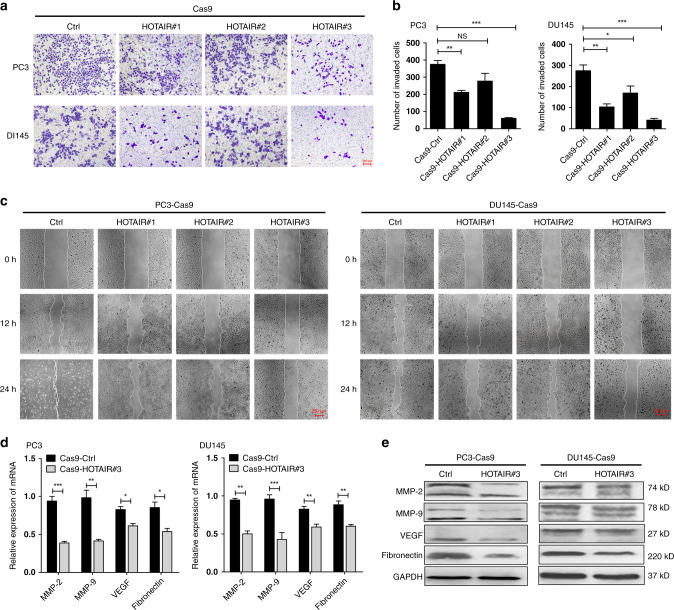
HOTAIR deficiency substantially reduces the invasiveness and metastasis of PCa cells. **a**, **b** Transwell assays revealed that transfection with Cas9-HOTAIR#3 markedly reduced cell invasiveness. Data are shown as the mean ± SD (*n* = 3), NS, not significant, *P < 0.05, ***P < 0.001. **c** Wound healing assays verified that deletion of HOTAIR notably inhibited PCa cell migration. **d**, **e** HOTAIR deletion reduced the mRNA and protein expression of metastasis-related genes. Data are shown as the mean ± SD (*n* = 3), *P < 0.05, **P < 0.01, ***P < 0.001.

Consistent with the aforementioned results, the invasion and metastasis of PCa cells transfected with the Cas9-HOTAIR#3 plasmid was markedly reduced compared with those of the Cas9-Ctrl and Cas9-HOTAIR #1 and #2 groups (Fig. 3a–c). RT-PCR confirmed that in PCa cells, deletion of HOTAIR could decrease the mRNA and protein levels of the metastasis-associated genes MMP-2, MMP-9, VEGF and fibronectin (Fig. 3d, e). These results suggest the involvement of HOTAIR in PCa cell migration.

### HOTAIR recruits PRC2 to regulate the expression of hepaCAM via H3K27me3 in PCa cells

Co-IP assays verified that EZH2, SUZ12 and H3K27me3 interacted in PC3 cells. With the Cas9 plasmid targeting HOTAIR, this correlation was attenuated, and the H3K27me3 levels were reduced (Supplementary Fig. 1a, b, *p* < 0.01). We inferred that this methylation process was mediated by HOTAIR; thus, PC3 cells were treated with two different inhibitors, DZNeP and the CPI-169 racemate, which targets PRC2 activity. Compared with that of the control group, the mRNA and protein expression of hepaCAM in the experimental group was significantly reversed (Supplementary Fig. 1c, d, *p* < 0.01), and the level of H3K27me3 was notably decreased (Supplementary Fig. 1d, *p* < 0.01).

### PRC2 is recruited to the promoter region of hepaCAM to induce methylation

**Fig. 4:**
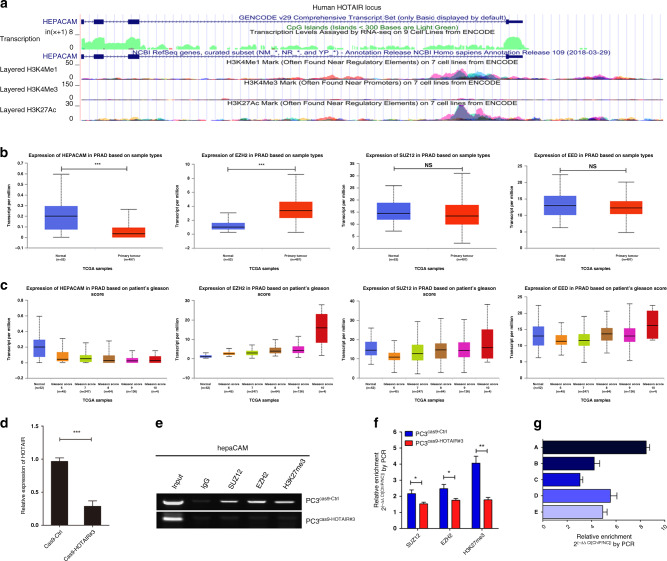
PRC2 is recruited to the hepaCAM promoter region to trigger methylation. **a** The human hepaCAM locus is shown by the UCSC Genome Browser. **b** TCGA database analysed the differential mRNA expression of hepaCAM, EZH2, SUZ12 and EED in PCa at various stages and normal tissues; ***P < 0.001. **c**–**f** ChIP-PCR suggested that HOTAIR-regulated PRC2 inhibited the expression of hepaCAM via H3K27me3. Data are shown as the mean ± SD (*n* = 3), NS, *n*ot significant, *P < 0.05, **P < 0.01, ***P < 0.001. **g** HOTAIR recruited PRC2 to a specific hepaCAM promoter region.

To investigate the potential mechanism of hepaCAM silencing, we focused on the hepaCAM gene locus and found that histone H3K27Ac and H3K4me1 were enriched near the transcription start site of hepaCAM, while in the promoter region, no such changes were observed (Fig. 4a). Therefore, we hypothesised that the silencing of hepaCAM may be related to other histone modifications in the promoter region. Through searching a TCGA database repository of Ualcan, we found a transcriptional profile of 52 normal prostate tissues and 491 PCa specimens by microarrays, which revealed that the expression of hepaCAM in the PCa tissues was markedly lower than in the normal tissues, while the mRNA level of EZH2 showed the opposite pattern (Fig. 4b). Moreover, the differential expression was closely related to the Gleason score (Fig. 4c). Unexpectedly, the expression levels of SUZ12 and EED were not significantly different (Fig. 4b, c). These data indicated that abnormal expression of hepaCAM and EZH2 might be related to the progression of PCa, and SUZ12 and EED function by being recruited to form a complex rather than through changes at the mRNA level.

ChIP-PCR assays showed that the chromatin fragments enriched by H3K27me3, EZH2 and SUZ12 monoclonal antibodies all contained hepaCAM, and the results were negative with HOTAIR knockout (Fig. 4d–f, *p* < 0.05). The promoter of hepaCAM was predicted to be located near −1700 bp or −356 to −606 bp. Therefore, we designed five pairs of primers in the range of −275 to −1919 bp. RT-PCR showed that fragment A (−275 ~ −674 bp) showed greater enrichment of hepaCAM (Fig. 4g), indicating that the PRC2 recruited by HOTAIR was located in this region.

### The reduced invasiveness and metastasis caused by HOTAIR deletion in PCa cells is related to hepaCAM re-expression

**Fig. 5:**
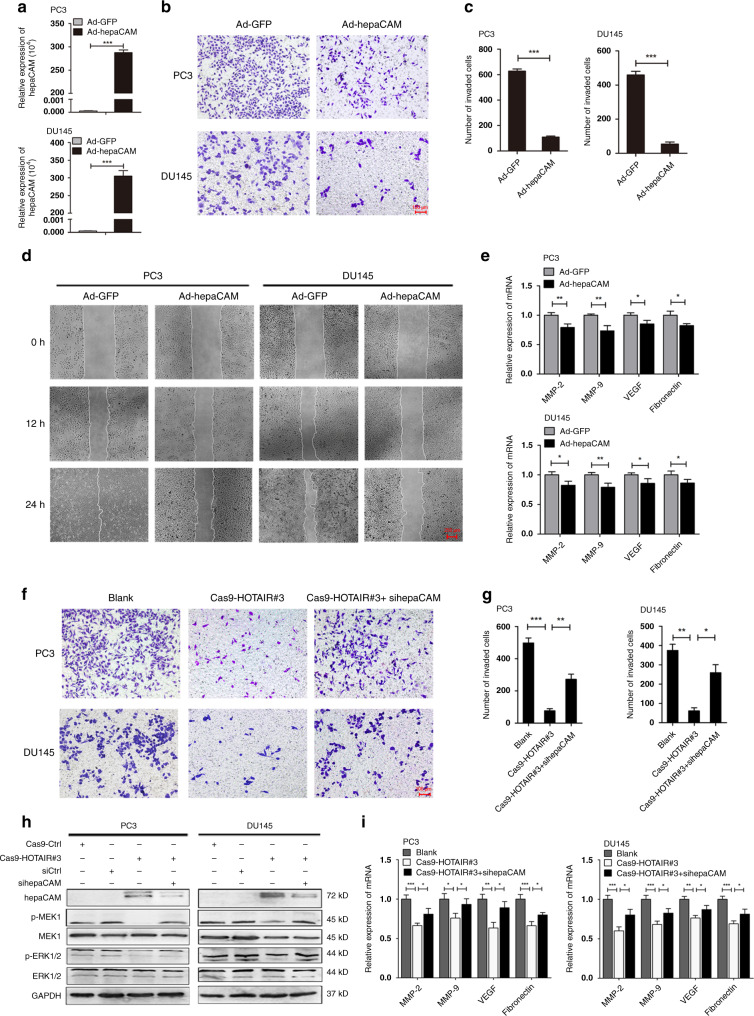
The reduced invasiveness and metastasis caused by HOTAIR deletion was related to hepaCAM re-expression. **a**–**c** Transwell assays indicated that overexpression of hepaCAM remarkably inhibited PCa cell invasiveness; ***P < 0.001. **d** The inhibited cell metastatic capabilities with hepaCAM re-expression. **e** mRNA expression of metastasis-associated genes; *P < 0.05, **P < 0.01. **f**, **g** HOTAIR deletion combined with hepaCAM knockdown diminished the inhibitory effect of HOTAIR on invasiveness. Data are shown as the mean ± SD (*n* = 3), NS, not significant, *P < 0.05, **P < 0.01, ***P < 0.001. **h** MEK/ERK signalling was limited with HOTAIR deletion but was reactivated if the expression of hepaCAM was also inhibited. **i** The expression of invasion-related genes; *P < 0.05, **P < 0.01, ***P < 0.001.

We further assessed the effect of HOTAIR silencing on the invasive and metastatic capacities of PCa cells. Overexpression of hepaCAM (Fig. 5a) substantially inhibited the invasion and metastasis of PCa cells (Fig. 5b–d) and significantly downregulated the expression of invasion-related genes (Fig. 5e). Knockout of HOTAIR notably diminished the invasiveness of PCa cells, but if hepaCAM was simultaneously knocked down by siRNA, this inhibitory effect was weakened (Fig. 5f, g, *p* < 0.01).

To evaluate the effect of HOTAIR on the MAPK signalling pathway, we detected the expression of signalling markers in PCa cells among the four groups by western blotting. We found that HOTAIR-depleted PCa cells showed upregulation of hepaCAM and downregulation of p-MEK1 and p-ERK1/2 but unchanged total MEK1 and ERK1/2 (Fig. 5h). The regulatory effect was weakened when hepaCAM was simultaneously knocked down. Furthermore, the absence of HOTAIR downregulated the expression of invasion-related genes but was had no effect when hepaCAM was also knocked down (Fig. 5i). Thus, HOTAIR enhances the invasiveness and metastasis of PCa by decreasing the expression of hepaCAM and activating the MEK/ERK signalling pathway.

### HOTAIR activates the MAPK pathway by inhibiting hepaCAM in PCa cells

We further explored the relationship between MAPK signalling and hepaCAM in cancer migration. PCa cells were treated with Ad-hepaCAM and S1P (activator of MEK/ERK). Overexpression of hepaCAM significantly diminished the invasiveness of PCa cells, while S1P notably recovered the invasive capability compared to that of the control group (Supplementary Fig. 2a, b). Re-expressing hepaCAM inhibited MEK/ERK signalling by inhibiting phosphorylation rather than the mRNA level (Supplementary Fig. 2c, d). The effect of downregulation of hepaCAM on invasion-associated genes was abolished by S1P (Supplementary Fig. 2c, d), suggesting that the inactivation of MAPK signalling regulated by hepaCAM substantially affected metastasis of PCa cells.

### DZNeP targeting improves the efficacy of docetaxel on migration

**Fig. 6:**
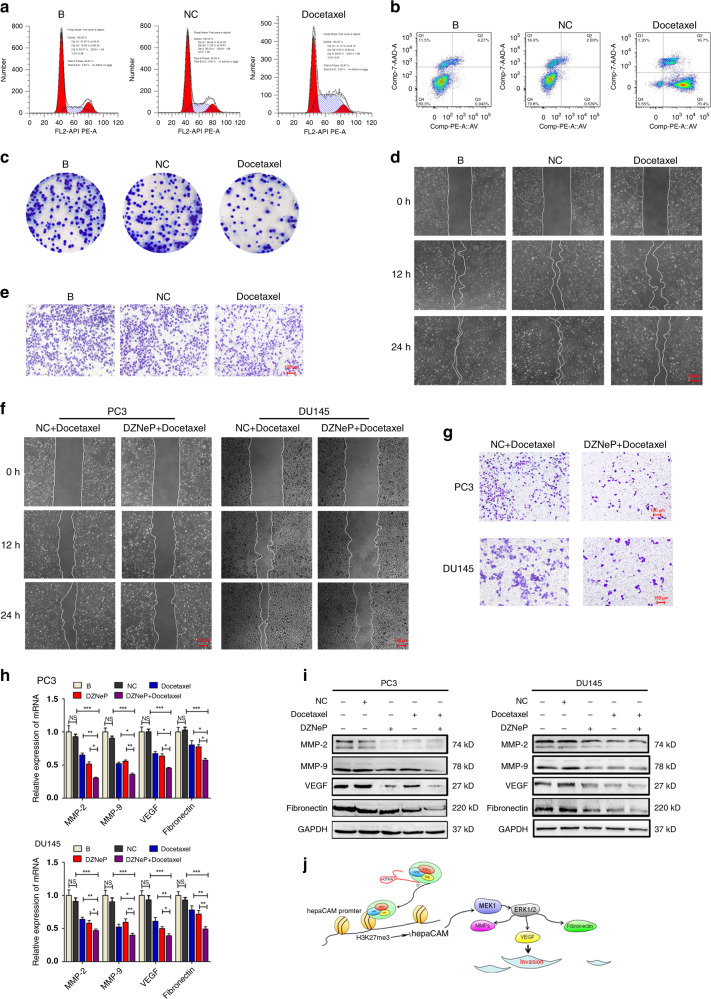
DZNeP targeting improves the efficacy of docetaxel on inhibition of migration. **a** The cell cycle arrest induced by docetaxel. **b** Influence of docetaxel on apoptosis of PC3 cells. **c** Docetaxel altered the proliferation of PC3 cells. **d**, **e** Changes in PC3 cell migration and invasion under docetaxel treatment were determined by wound healing assay and transwell assays. **f**, **g** Docetaxel combined with DZNeP inhibited the migration and invasion of PC3 and DU145 cells. **h**, **i** Effects of docetaxel combined with DZNeP on the expression of migration-related genes. **j** Model depicts the role of HOTAIR during the invasion and metastasis of PCa. HOTAIR recruits PRC2 to the specific promoter sites of hepaCAM, leading to higher H3K27me3 level and decreased hepaCAM expression. Downregulation of hepaCAM results in decreased inhibitory effect on MAPK signalling and enhanced PCa migration. Data are shown as the mean ± SD (*n* = 3), NS, not significant, *P < 0.05, **P < 0.01, ***P < 0.001.

To evaluate the therapeutic relevance of this finding, we assessed whether DZNeP could enhance the inhibitory effect of docetaxel on metastasis in PCa cells. Docetaxel blocked the cell cycle of PCa cells in the S phase (Fig. 6a), significantly accelerated early apoptosis (Fig. 6b), and notably inhibited cell proliferation (Fig. 6c). However, the inhibitory effect of docetaxel on migration was not obvious (Fig. 6d, e). DZNeP has been verified to have inhibitory effects on various cancers, including PCa, without affecting the growth of normal cells. Here, we treated PC3 and DU145 cells with DZNeP in combination with docetaxel. Wound healing and transwell assays showed that DZNeP enhanced the inhibitory efficacy of docetaxel in cell invasion and metastasis (Fig. 6f, g). Combined DZNeP and docetaxel were associated with robust inhibition of the mRNA and protein expression of genes involved in cancer migration (Fig. 6h, i). Taken together, these data demonstrate that DZNeP can enhance the efficacy of docetaxel on invasiveness and metastasis in prostate cancer.

## Discussion

The long noncoding RNA HOTAIR has been found to be exclusively expressed in tumours. Recent studies have confirmed that HOTAIR expression is closely related to the invasion, migration and prognosis of malignant tumours.^22–24^ It is believed that HOTAIR elicits its effect by silencing the expression of target genes through epigenetic modification.^9^ In this study, we found that HOTAIR was highly expressed in PCa by analysing a large number of patient samples from TCGA dataset. We also demonstrated that the expression of HOTAIR increased in a clinical stage-dependent manner in the blood of the PCa patients. We showed that deletion of HOTAIR could significantly inhibit the invasion and metastasis of PCa cells. These reports indicated the unexpected ability of lncRNAs to induce tumour migration.

Accumulating studies have shown that silencing of suppressors and abnormal activation of oncogenes in tumours are caused by epigenetic modifications during carcinogenesis.^25^ Histone modification and noncoding RNA regulation are two important regulatory mechanisms.^26,27^ HepaCAM is an Ig-like adhesion molecule that can be detected in normal tissues and is usually absent in cancer tissues.^15,16^ The absence of cell adhesion molecules allows tumour cell detachment and dissemination from the primary mass. HepaCAM has been proven to have anti-cancer properties in various tumours.^28,29^ Moreover, we found that the CpG island of hepaCAM was hypermethylated and that demethylation drugs can reverse its expression, but the mechanism remains to be further investigated.^30,31^ Therefore, in this study, we aimed to further explore the silencing mechanism of hepaCAM in tumours and to provide a new treatment programme. We revealed that HOTAIR could recruit PRC2 to the promoter region of hepaCAM, and EZH2 exerted histone-lysine N-methyltransferase activity, resulting in higher H3K27me3 and leading to hepaCAM silencing. Therefore, we concluded that hepaCAM acts as a key bridge molecule in the process of distant metastasis that is altered by HOTAIR in prostate cancer. These results were distinct and identified a new function for HOTAIR in inhibiting tumour hepaCAM by targeting the promoters. This is the first demonstration of the link between HOTAIR and hepaCAM from an epigenetic perspective and an explanation of their regulatory mechanisms in PCa metastasis.

The MAPK signalling pathway has been proven to be abnormally activated in various malignant tumours and closely related to tumour proliferation, invasion, metastasis and drug resistance.^32–34^ Previous studies verified ERK as a downstream target gene of hepaCAM that plays a key role in the metastasis of PCa by affecting ECM degradation and neovascularisation in malignant tumours.^35^ Here, we found that HOTAIR promotes invasion and metastasis by activating the MEK/ERK axis with inhibited expression of hepaCAM.

Docetaxel, a first-line chemotherapeutic drug for the treatment of advanced prostate cancer, effectively inhibits PCa progression by forming a stable non-functional vascular bundle, causing cancer cell arrest and apoptosis in the G2/M phase. However, the inhibitory effect of docetaxel on cancer migration is not optimal. Here, we found that high expression of HOTAIR was related to adverse clinical features of PCa and associated with tumour insensitivity to docetaxel. As a PRC2 inhibitor, DZNeP has been proven to induce anti-leukaemic effects by targeting EZH2 in AML.^36^ Here, we found that the migration of PCa cells was significantly inhibited by the combination of DZNeP and docetaxel. We identified a novel mechanism in PCa cells in which PRC2 recruited by HOTAIR mediated the resistance to docetaxel. Targeting PRC2 could significantly improve the sensitivity of PCa cells to chemotherapy.

Our findings clearly imply that HOTAIR functions as an oncogene in downregulating the tumour suppressor gene hepaCAM by targeting histone modification at specific sites in the promoter. Further experiments indicated that the absence of hepaCAM contributed to modulating PCa cell migration through distinct pathways (Fig. 6j). These findings uncovered a novel compensatory mechanism for uncontrolled invasion and metastasis when cancer cells become insensitive to docetaxel. This project has exploratory and clinical application prospects.

## Supplementary information

Supplementary Material

## Data Availability

The datasets used and/or analysed during the current study are available from the corresponding authors on reasonable request.
