# Supplementary material for: Long noncoding RNA HOTAIR regulates the invasion and metastasis of prostate cancer by targeting hepaCAM
Source: Br J Cancer. 2020 Oct 7;124(1):247–58. doi: 10.1038/s41416-020-01091-1 (PMC7782544; doi:10.1038/s41416-020-01091-1)
Supplement: Supplementary file 1 — Supplementary Material [file 41416_2020_1091_MOESM1_ESM.doc]

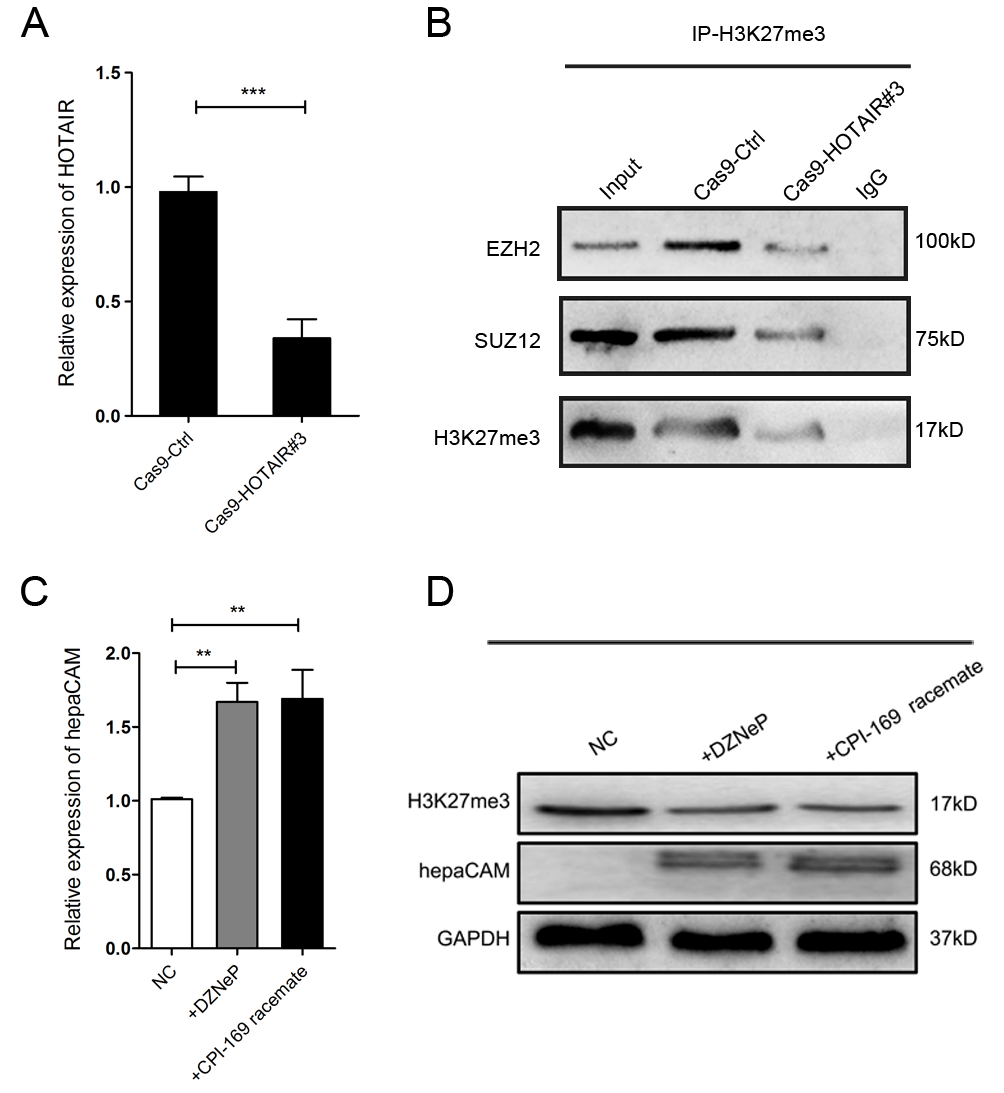


Supplementary Fig. 1 HOTAIR recruits PRC2 to down-regulate hepaCAM via H3K27me3 in PCa cells. a, b Co-IP assay verified the interacted of EZH2, SUZ12 and H3K27me3 in PC3 cells; ***P < 0.001. c, d Both DZNeP and CPI-169 racemate significantly downregulated the expression of HOATIR and H3K27me3 and reversed the expression of hepaCAM. Data were shown as mean ± SD (*n* = 3), **P < 0.01.


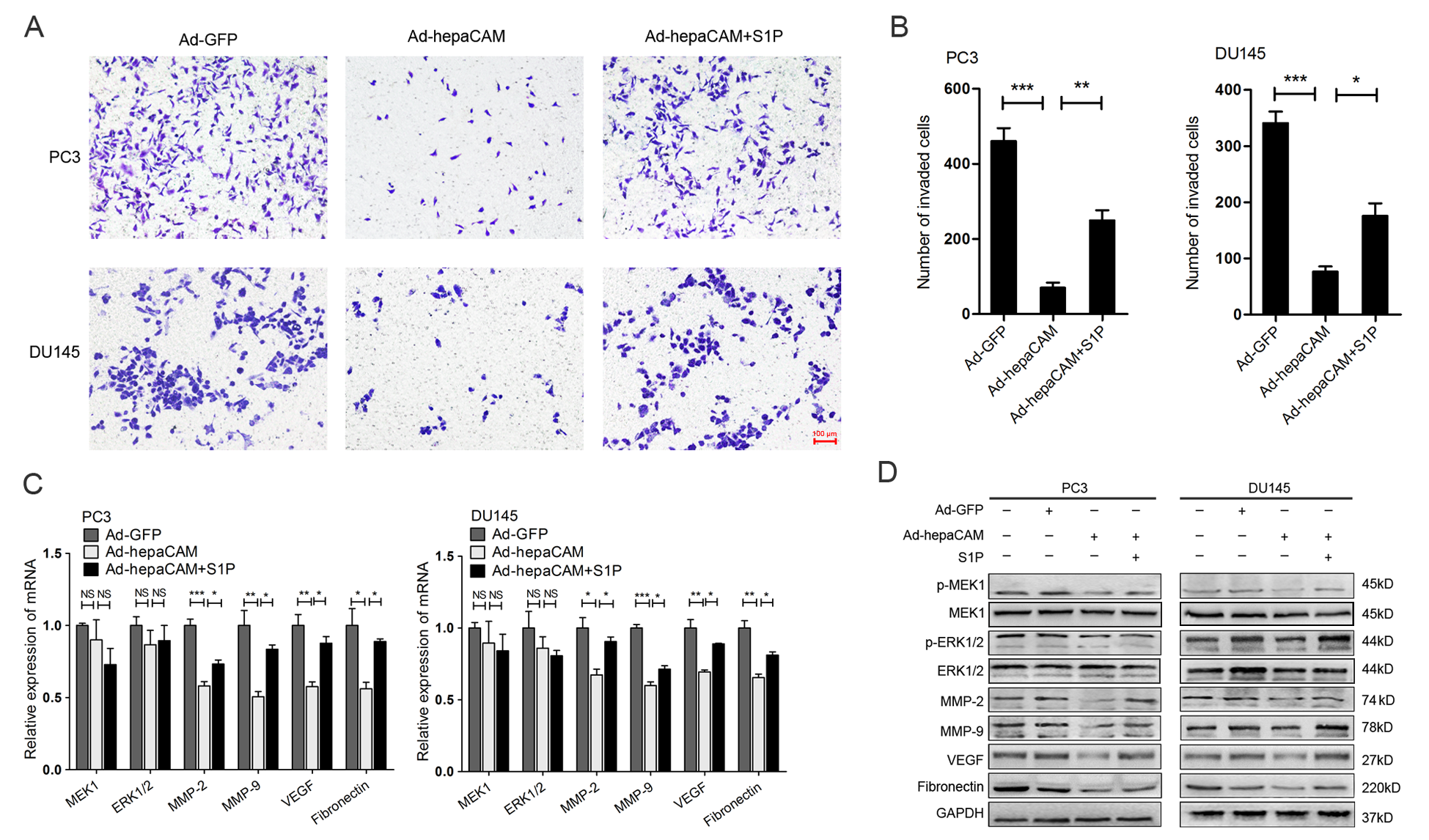
Supplementary Fig. 2 HOTAIR activate MAPK pathway by inhibiting hepaCAM in PCa cells. a, b The PCa cell regained invasiveness as the activation of MAPK signaling; *P < 0.05, **P < 0.01, ***P < 0.001. c The mRNA and protein level of metastasis-related genes. Data were shown as mean ± SD (*n* = 3), NS, not significant, *P < 0.05, **P < 0.01, ***P < 0.001.
